# *Bacillus*-based inoculants enhance drought resilience in soybean: agronomic performance and remote sensing analysis from multi-location trials in Brazil

**DOI:** 10.3389/fpls.2025.1630127

**Published:** 2025-07-23

**Authors:** Julio Cezar Souza Vasconcelos, Caio Simplicio Arantes, Eliane Aparecida Gomes, Christiane Abreu de Oliveira-Paiva, Sylvia Morais de Sousa, Eduardo Antonio Speranza, João Francisco Gonçalves Antunes, Ubiraci Gomes de Paula Lana, Geraldo Magela de Almeida Cançado

**Affiliations:** ^1^ Fundação de Apoio à Pesquisa e ao Desenvolvimento (FAPED), Campinas, São Paulo, Brazil; ^2^ Embrapa Maize and Sorghum, Sete Lagoas, Minas Gerais, Brazil; ^3^ Embrapa Digital Agriculture, Campinas, São Paulo, Brazil

**Keywords:** *Glycine max* (L) merrill., bioinoculants, drought tolerance, statistical modeling, vegetation index, spectral analysis

## Abstract

Climate change exacerbates drought stress, posing challenges to global soybean grain yield. This study assesses the effectiveness of microbial inoculants derived from two *Bacillus velezensis* (strains 5D5, 6E9) and one *Bacillus subtilis* (strain 1A11), which were previously selected in vitro to promote growth and enhance drought resilience in soybeans (*Glycine max* [L.] Merr.), and evaluated through agronomic metrics and remote sensing. A greenhouse experiment was conducted to evaluate the performance of these inoculants under both irrigated and drought conditions. The inoculants were applied at the dose-range of 1, 2, 3 and 4 mL Kg-1 of seed to identify the optimal dose. The greenhouse results indicated that for many tested doses, the three bacterial strains significantly increased shoot fresh weight, shoot dry weight, and root dry weight compared to control treatments. Multi-location field-trials in Brazil (Birigui, Itapira and Piracicaba) were conducted during the growing seasons of 2022–2023 and 2023–2024, using 3 mL Kg-1 of seed as a reference dose. These field-trials revealed yield improvements of 11.3 to 18\% for inoculated treatments, with *B. subtilis* 1A11 achieving the highest grain yield of 620 Kg ha-1 over the control. However, all three microbial inoculants significantly enhanced soybean development and grain yield relative to non-inoculated controls. Vegetation indices, particularly the Enhanced Vegetation Index 2 (EVI2), derived from PlanetScope satellite and Unmanned Aerial Vehicle (UAV) imagery, demonstrated a high overlap between field data and model predictions, confirming the value of remote sensing as a predictive tool. Climatic variability significantly impacted the yield in field-trials, with 2022–2023 (4.28 t ha-1 outperforming 2023–2024 (3.34 t ha-1) due to higher temperatures (>40 °C) and lower rainfall in the last season. Meanwhile, locations with balanced precipitation, like Itapira, showed superior grain yield. Statistical modeling confirmed inoculant efficacy and EVI2's utility in production measurement. This study emphasizes that microbial inoculants can serve as sustainable strategies to mitigate the impacts of drought. By integrating *Bacillus*-based bioinoculants into soybean cultivation and utilizing both agronomic and remote sensing metrics for validation, we can enhance resilience and ultimately support food security amid climate variability.

## 1 Introduction

Many of the world’s agricultural regions are experiencing increasingly intense changes in climate patterns. These changes have affected the intensity and regularity of rainfall, caused significant fluctuations in historical temperature averages, and intensified the occurrence of pests, diseases, and various harmful abiotic effects during the crop cycle (Tchonkouang et al., 2024).

To mitigate the negative impacts of these environmental challenges, various strategies have been implemented to enhance the adaptive capacity of agricultural species. One approach involves incorporating genetic traits linked to stress tolerance into breeding programs, enabling plants to better withstand environmental limitations. Another strategy focuses on modifying the production environment to optimize conditions for crop growth. However, these interventions can sometimes compromise ecosystem sustainability. Fortunately, sustainable and economically viable alternatives are emerging that can be integrated into existing production systems without causing environmental harm. These alternatives not only support crop development but also maintain or even enhance productivity, contributing to global food security. The use of microorganisms in association with cultivated plants has become a promising alternative to conventional agricultural inputs (Singh et al., 2011). Commercially available products include biofertilizers as substitutes for inorganic fertilizers, biopesticides as alternatives to synthetic pesticides, and various biostimulants that promote plant growth (O’Callaghan et al., 2022). These microbial-based solutions offer a sustainable approach to improving crop performance while reducing reliance on chemical inputs.

Soybean (*G. max*) is a crop that particularly benefits from microbiological inoculants, which enhance field performance and grain yield (Schmidt et al., 2015). A key example is biological nitrogen fixation (BNF), a process mediated by diazotrophic bacteria of the genus *Bradyrhizobium* spp. These bacteria form symbiotic relationships with leguminous plants, including soybeans, enabling the conversion of atmospheric nitrogen into a biologically usable form. This process reduces the need for synthetic nitrogen fertilizers, offering both economic and environmental benefits. Brazil has been a pioneer in adopting this technology, with estimates suggesting annual savings of approximately US$15 billion due to the replacement of nitrogen fertilizers with biologically fixed nitrogen (Döbereiner, 1997; Hungria and Mendes, 2015; Hungria and Nogueira, 2019).

Several studies have demonstrated that plant growth-promoting bacteria (PGPB) provides numerous direct and indirect benefits to crop plants (Sarma and Saikia, 2014; Rho et al., 2018). These microorganisms are known to establish close associations with host plants, contributing significantly to enhanced stress tolerance, including drought (Pandey et al., 2016; Zhang et al., 2019; Dubey et al., 2021; Liu et al., 2025). Among the various PGPB, species belonging to the genus *Bacillus* are of particular interest due to their ability to form endospores, which enables them to survive under extreme abiotic stresses such as temperature and pH, radiation, desiccation, ultraviolet exposure, and pesticide contamination (Bahadir et al., 2018). For instance, *B. velezensis* has been shown to facilitate the establishment of a specific symbiosis between soybean and *Bradyrhizobium* strains, as well as to promote root development under water-limited conditions (Kondo et al., 2025). In rice, *B. velezensis* contributes to improved drought tolerance by reducing the accumulation of reactive oxygen species (Park et al., 2024). Recent studies also have demonstrated that *B. subtilis* plays a significant role in enhancing plant drought tolerance. It primarily alleviates drought stress by secreting various metabolites and modulating plant hormone levels (Azeem et al., 2023; Kuramshina and Khairullin, 2023; Ren et al., 2025). Furthermore, *B. subtilis* promotes root development and improves the soil microbial environment, thereby increasing water uptake efficiency (Khan et al., 2024) and crop productivity (Ren et al., 2025).

According to Bettiol (2022), bioproducts used in agriculture are classified into three main categories: biofertilizers, biostimulants, and biopesticides. In this study, we evaluated the efficacy of three *Bacillus*-based microbiological inoculants applied as biostimulants to soybean seeds at the time of sowing. The aim was to assess their potential to mitigate developmental and yield constraints under real field conditions. In addition to traditional agronomic evaluation methods, we employed remote sensing technologies, including drone and satellite imagery, to calculate vegetation indices (VIs). These indices were used to monitor crop development and correlate with agronomic performance metrics.

The integration of remote sensing with agricultural practices has become a widely used tool in the field, including the evaluation of irrigation practices on the field (Ozdogan et al., 2010; Bégué et al., 2018). The use of images obtained from UAVs and satellites has enabled the development of increasingly accurate predictive production models. Vasconcelos et al. (2025) and Vasconcelos et al. (2023), while working with sugarcane crops and temporal images obtained via satellites and drones, developed models based on VIs capable of predict sugarcane production months ahead of the harvest date. This capability offers numerous advantages for sugarcane growers, mills, and the overall production chain associated with this crop. Similarly, Santos et al. (2024), utilizing aerial images captured by drones in sugarcane experimental fields, demonstrated the effectiveness of a phosphate-solubilizing microbiological inoculant compared to traditional cultivation methods that do not use the inoculant. Particularly for soybean crops, recent studies have shown the advantages of using remote sensing to monitor the production cycle and estimate grain yield (Prince Czarnecki et al., 2025). Hu et al. (2023) used images in the visible *spectrum* collected by UAV and field data to develop a high-accuracy model based on machine learning to estimate vegetation cover fraction, chlorophyll content and breeding maturity. Joshi et al. (2024) developed a comparative study of several artificial intelligence (AI) models for predicting soybean grain yield, based on high spatial resolution multispectral images obtained from PlanetScope nanosatellites. The results showed that combinations of images obtained at different growth stages help to increase the accuracy of models evaluated. Amaral et al. (2024) used a machine learning model based on random forests and multispectral images from the Sentinel-2 satellite to define more appropriate vegetation indices to identify the spatiotemporal variability of vegetation vigor and canopy structure of soybean plants.

This study investigated the effectiveness of three bacterial inoculantss — *B. velezensis* (strains 5D5 and 6E9) and *B. subtilis* (strain 1A11) — in increasing the grain yield of soybean (*G. max*) when applied to seeds before sowing. The agronomic performance was assessed using grain yield metrics and VIs, with the results compared to non-inoculated controls to quantify the effects of the treatments.

## 2 Materials and methods

### 2.1 Isolation, selection, characterization and identification of bacterial strains

To carry out this study, strains of the bacteria *B. velezensis* (5D5 and 6E9) and *B. subtilis* (1A11) were isolated and selected *in vitro* based on their potential to promote growth in plants, as well as their ability to survive under osmotic stress as described by de Oliveira-Paiva et al. (2024). The microorganisms were isolated from soil samples, starting with the screening of a total of 414 strains samples. In the subsequent stages of evaluation and screening, which included the capacity to grow in medium with a high concentration of sorbitol, assessing exopolysaccharide (EPS) production capacity, biofilm production, and a qualitative evaluation of siderophore and growth phytoregulators, the three strains identified as 5D5 (germplasm code BRM051757), 6E9 (germplasm code BRM051761), and 1A11 (germplasm code BRM051734) were selected for further analysis. The methods and protocols used during this stage of the study are outlined below.

#### 2.1.1 Identification of *Bacillus* strains

Bacterial genomic DNA was extracted with the Wizard Genomic DNA Purification Kit (Promega, USA) and 16S rRNA gene amplified with the primers 8F and 1492R (Turner et al., 1999). PCR reactions were performed with 30 ng of bacterial genomic DNA, 2.5 µL 10X PCR buffer (20 mM Tris–HCl pH 8.4, 50 mM KCl), 0.4 *µ*M of each primer, 100 *µ*M dNTP, 2.5 mM MgCl_2_, and 1 U Taq DNA Polymerase (Invitrogen, USA) in a total volume of 25 *µ*L. PCR was performed with the following conditions: 95°C for 2 min, 30 cycles of 30 s at 94°C, 30 s at 55°C and 2 min at 72°C. Finally, reactions were incubated for 10 min at 72°C. The amplification products were purified with the ExoSAP-IT Kit (USB, USA), and sequenced with the primers 8F, 1492R, 515F (Turner et al., 1999), and 902R (Hodkinson and Lutzoni, 2009) using Big Dye Terminator v3.1 kit (Applied Biosystems, USA). The samples were analyzed in the automated sequencer ABI PRISM 3500 XL Genetic Analyzer (Applied Biosystems, USA), and DNA sequences were compared with the GenBank DNA sequence database using the BlastN program (Altschul et al., 1997).

#### 2.1.2 Bacterial strains selection

To conduct this study, the bacterial strains 5D5 (BRM051757) and 6E9 (BRM051761) of *B. velezensis*, as well as strain 1A11 (BRM051734) of *B. subtilis*, were isolated and characterized by Embrapa Maize and Sorghum´s research group. The strains were obtained from soil samples collected in the Caatinga biome, a semi-arid region located in Ceará, Brazil, and were deposited in the Collection of Multifunctional and Phytopathogenic Microorganisms at Embrapa Maize and Sorghum. Experimental procedures to assess *in vitro* plant growth-promoting properties and drought stress tolerance were conducted at the Embrapa Maize and Sorghum research facilities.

#### 2.1.3 Bacterial growth in medium with high concentration of sorbitol

The strains were inoculated onto tryptone soy agar (TSA; g L^−1^: tryptone 15, papain-digested soybean 5, NaCl 5, agar 15) enriched with 10% (w/v) sorbitol at concentrations of 520 and 780 g L^−1^, corresponding to water activities (aw) of 0.897 and 0.807, respectively. Plates were incubated at 40°C for 72 hours.

#### 2.1.4 Exopolysaccharide, biofilm and siderophore production

EPS production was evaluated following Paulo et al. (2012). Sterile filter paper discs (5 mm Ø) were placed in Petri dishes containing the culture medium described by Guimarães et al. (1999) and inoculated with 5 *µ*L of each bacterial isolate grown in TSB. Plates were incubated at 30°C for 24 h, and EPS production was indicated by a mucoid colony surrounding the discs. Production was confirmed by mixing the mucoid substance with 2 mL of absolute ethanol: a precipitate indicated positive EPS production, while turbidity indicated a negative result. Biofilm formation was assessed using the spectrophotometric method described by Stepanović et al. (2007). Qualitative siderophore production analysis was performed as previously described by Ribeiro et al. (2018) and Arora and Verma (2017). Absorbance was measured at 630 nm using a Perkin Elmer spectrophotometer (USA), and siderophore production was estimated following the method described by Ribeiro et al. (2018).

#### 2.1.5 IAA-like molecules production

Each strain was grown in a TSB medium supplemented with 1.0 mg mL^−1^ tryptophan as an IAA precursor and incubated at 30°C for five days at 100 rpm in the dark (Ribeiro et al., 2018). After centrifugation at 4250 × g for 10 min, 0.1 mL of the supernatant was mixed with 0.1 mL of the Salkowski reagent (Loper and Schroth, 1986) and incubated for 20 min in the dark. The concentration of IAA-like molecules in the supernatant was determined in triplicate by the colorimetric measurement at 540 nm and compared to a standard curve prepared from commercial IAA at concentrations ranging from 0 to 100 *µ*g mL^−1^ (Patten and Glick, 1996).

### 2.2 Greenhouse experiments and plant material

The greenhouse experiments were carried out at Embrapa Maize and Sorghum, located in Sete Lagoas, MG, Brazil, in 2023. The study evaluated the dose-range effect of inoculants individually formulated with the strains 1A11, 5D5, and 6E9 on the growth of soybean seedlings. This assessment was carried out under conditions of adequate moisture availability, as well as during episodes of drought stress.

The inoculants were prepared with bacterial colony-forming unit concentration for each strain as follows: 1A11 = 8.67x10^9^ bacterials mL^−1^; 5D5 = 1.62x10^10^ bacterial mL_-1_ to CFU; and 6E9 = 9.41x10^9^ bacterial mL_-1_ to CFU. Soybean seeds were treated with doses of 1, 2, 3, and 4 mL kg^−1^ for all tested strains, with application occurring just before sowing. The soybean seeds were homogenized with the liquid inoculant by mixing them in a container until all seeds were thoroughly moistened and covered by the inoculant. As a standard practice for soybean cultivation to enhance symbiotic nitrogen fixation, it was applied to all treatments the liquid inoculant SimbioseNod™, formulated from strains SEMIA 5079 and SEMIA 5080 of *Bradyrhizobium japonicum*, at a concentration of 7.2 x 10^9^ bacterial mL_-1_ to CFU of inoculant, at a dose of 3 mL kg^−1^ of seed. The *B. japonicum* inoculant was applied separately, immediately after the tested *Bacillus* inoculants were administered. The control group received only the *B. japonicum* and this inoculant was mixed with the seed mass in a manner similar to the process used for the *Bacillus* inoculants.

For each experimental plot, 20 L pots filled with clayey red latosol, typical of the Brazilian Cerrado biome, were utilized. Soil fertilization and liming were consistent across all treatments, adhering to recommendations based on soil physicochemical analysis. Two soybean plants of the cultivar ‘BRS Valiosa RR’, were maintained in each pot. The experimental design was completely randomized, comprising two sets of 26 treatments with three replicates per treatment. The data were analyzed by the R statistical package.

Of the 78 experimental units, 39 were sustained under adequate irrigation, with soil field capacity continuously maintained at -18 kPa. The other 39 experimental units underwent drought stress conditions starting 27 days after germination, simulated by keeping the soil at a water tension of -138 kPa for 14 days. Daily monitoring of soil water availability for both irrigated and non-irrigated treatments was conducted using a tensiometer installed at a depth of 20 cm in each pot. At the conclusion of the drought stress period, the irrigated and non-irrigated plants were collected at the early flowering stage (R1), and the weights of fresh and dry matter for both the aerial parts and roots were evaluated.

### 2.3 Field-trials and plant material

To assess the impact of microbiological inoculants on soybean crops, field-trials were established in three different locations in Brazil: Birigui (geographical coordinate -21.318220, -50.355165), Itapira (-22.401927, -46.778187) and Piracicaba (-22.769027, -47.582643) and evaluated over two consecutive growing seasons (2022–2023 and 2023–2024). The soybean varieties was chosen based in its agronomic recommendation for each region and in its precocity of the cycle. In Birigui was cultivated the variety ‘BMX Nexus i2X’ of the maturity group 6.4, in Itapira was cultivated the variety ‘Monsoy 6601 i2X’ of the maturity group 6.6, and in Piracicaba was cultivated the variety ‘NS 5933 iPro’ of the group of maturity 6.1. To establish a stand of approximately 260,000 plants per hectare, about 13 seeds were sown per linear meter of furrow, maintaining a spacing of 50 cm between rows and the planting depth was set at 5 cm. During the planting of seeds was applied granulated fertilizer with a composition of 3-21-21 (NPK, YaraBasa™) at rates based on the soil’s chemical analysis for each location: 150 kg ha^−1^ in Birigui; 130 kg ha^−1^ in Itapira; and 180 kg ha^−1^ in Piracicaba and the fertilizer was positioned next to the seed, 5 cm below it. During the phenological phases between V3 and V6, a foliar micronutrient fertilizer (Micromax Foliar Concentrate™, Aqua do Brasil) was applied at a rate of 500 g ha^−1^.

The field-trials of Birigui and Itapira consisted of 16 experimental plots for each location and twenty rows of soybean measuring 0.5 m between furrows and 100 m in length, while the field-trial in Piracicaba also consisted of 16 experimental plots but with 10 rows of soybean measuring 0.5 m between furrows and 25 m in length. For each soybean variety, there were 4 blocks per field-trial, totaling 48 experimental plots per season and 96 experimental plots considering two harvest seasons. The random block design with four blocks per field-trial was adopted in this study to enable the measurement of random environmental variation. All experimental fields followed management practices aligned with standard commercial soybean cultivation in Brazil, ensuring consistent agronomic standards across all varieties. Soybean plots were harvested and measured in tons of soy per hectare (t ha^−1^) at the end of their growth cycle, once the seeds reached peak of maturation (13% of humidity based on the dry weight). Yield was assessed based on the average grain weight obtained from experimental replicates for each treatment across all locations (Birigui, Itapira and Piracicaba) over two consecutive growth cycles: 2022–2023 and 2023–2024.

### 2.4 Field inoculation

To assess the impact of three microbiological inoculants on soybean crops, each bacterial strain—5D5, 6E9, and 1A11—formulated as liquid inoculant was applied individually at a rate of 3 mL of liquid inoculant per kilogram of soybean seeds at the same concentration of the greenhouse study. A control treatment without inoculant application was included for comparison.

The inoculants containing strains 5D5, 6E9, and 1A11 were applied to the seeds in a similar manner to the procedure described in the greenhouse experiment. After application, the seeds were allowed to dry for five minutes before sowing, making sure to avoid direct sunlight exposure. Additionally, after the *Bacillus* inoculants application, *B. japonicum* inoculant was applied to the seeds of all treatments, including the control, using the same commercial product at the same concentration and dosage as in the greenhouse experiment.

### 2.5 Weather data

The climate data for the field-trials in Birigui, Itapira and Piracicaba were compiled from the NASA Power (NASA POWER Project, 2024) database as indicated by Monteiro et al. (2018). The dataset, which spans the entire soybean growing season for each location and harvest season, was sourced directly from the NASA POWER Project (2024).

### 2.6 Vegetation indices

The EVI2 VI (Jiang et al., 2008) was calculated for all field-trials during the phenological phase of vegetative growing. This VI can be used as an alternative to the traditional NDVI (Normalized Difference Vegetation Index) without changing the spectral bands used in its calculation (red and near infrared), which often reach saturation points in the most advanced stages of crop development, making it difficult to observe differences between treatments (Santos et al., 2024). For the fields of Birigui and Itapira were used satellite images from the PlanetScope PSB.SD instrument (Frazier and Hemingway, 2021), provided by RedeMAIS/MJSP source, with spatial resolution of 3 m/pixel and daily temporal resolution, regarding the following bands: blue (B): 465–515 nm; green (G): 547–583 nm; red (R): 650–680 nm; red edge (RE): 697–713 nm; and near-infrared (NIR): 845–885 nm. For the field of Piracicaba, due to the smaller size of the experimental area, were used images obtained from missions performed by a drone, model DJI Phantom 4 Pro equipped with RTK and two cameras: one RGB, for images in the visible *spectrum*; and another for multispectral array, covering the following bands: blue (B): 450 ± 16 nm; green (G): 560 ± 16 nm; red (R): 650 ± 16 nm; red edge (RE): 730 ± 16 nm; and near-infrared (NIR): 840 ± 26 nm. In this case, for each mission, orthomosaics were generated by the Agisoft Metashape software, considering the aforementioned bands with a ground sample distance (GSD) of approximately 10 cm. The satellite images or the orthomosaic assembled from drone images were then used to extract and calculate the VIs of the field-trials. For both cases, only the images or othomosaic that best represented the phenological *stadium* of vegetative growth (approximately 70 to 90 days) were effectively chosen to extract the EVI2 values. The average EVI2 value of the internal pixels of each experimental plot, excluding the pixels considered as borders, was then calculated and considered in this study.

### 2.7 Evaluation of variance inflation factor, data normality and homoscedasticity

In order to enhance the prediction model’s stability and address potential multicollinearity concerns, all predictor variables were incorporated into the initial analysis. The assessment of the Variance Inflation Factor (VIF) (Belsley et al., 1980) was performed to detect and eliminate variables with high collinearity, leading to a more streamlined and reliable selection of variables. The Shapiro-Wilk test was used for normality (Shapiro and Wilk, 1965) and the Breusch-Pagan test for homoscedasticity (Breusch and Pagan, 1979).

### 2.8 Variables definition

To investigate the determinants of soybean productivity, we define a set of explanatory variables that comprise both categorical and continuous covariates. The response variable is the grain yield, denoted by *y_i_*, measured in tons per hectare (t ha^−1^). Each observation *i* = 1*,…*,96 corresponds to a unique combination of experimental conditions. The variables are structured as follows:

- *y_i_*: Grain yield (t ha^−1^);
- *x_i_*_1_: Block factor (Block 1 to Block 4). Since it contains four levels, three binary (dummy) variables are created: *b_i_*_1_ *,b_i_*_2_ *,b_i_*_3_;
- *x_i_*_2_: Treatment factor (Control, 1A11, 5D5, and 6E9), represented using three dummy variables: *ti*1*,ti*2*,ti*3;
- *x_i_*_3_: Location factor (Birigui, Itapira, and Piracicaba), modeled with two dummy variables: *l_i_*_1_ *,l_i_*_2_;
- *x_i_*_4_: Year of cultivation (2022–2023 and 2023–2024), encoded by a single dummy variable: *w_i_*_1_;
- *x_i_*_5_: Total accumulated precipitation during the crop cycle (in millimeters), a continuous variable;
- *x_i_*_6_: Mean value of the Enhanced Vegetation Index 2 (EVI2) over the crop cycle, a continuous variable.

Categorical variables (*x_i_*_1_ to *x_i_*_4_) were transformed into binary indicators through dummy encoding to ensure compatibility with linear modeling frameworks. Continuous variables (*x_i_*_5_ and *x_i_*_6_) were retained in their original form to preserve their numeric information. This structure enables the application of linear regression models and facilitates the interpretation of individual effects on soybean yield.

### 2.9 Statistical model

A linear regression model was used to evaluate the effect of explanatory variables on soybean grain-yield, measured in tons per hectare (t ha^−1^). The model includes both categorical and continuous covariates previously described and is given by:


$$y_{i}=\beta_{0}+\sum_{j=1}^{p}\beta_{j}x_{ij}+\epsilon_{i},$$

where *y_i_* represents the response variable in observation *i*. The term *β*_0_ corresponds to the model intercept, which indicates the value of the response variable when all the explanatory variables are zero. The coefficients *β_j_* are the regression parameters associated with the explanatory variables *x_ij_*, for *j* = 1,2*,…,p*, which quantify the influence of each of these variables on the response variable. The variables *x_ij_* are the explanatory variables that can directly influence the response variable. Finally, the term *ϵ_i_* represents the random error associated with observation *i*, which captures the variations unexplained by the variables included in the model. It is assumed that the error *ϵ_i_* is a random variable with a normal distribution, mean zero, and constant variance, i.e., *ϵ_i_* ∼ *N*(0*,σ* ^2^). Thus, the equation expresses how the response variable is affected by the explanatory variables, incorporating an error component that reflects the unexplained variability.

The model was fitted using the lm() function from the R software environment (R Core Team, 2024), which estimates the regression coefficients using the Ordinary Least Squares (OLS) method. This method aims to minimize the sum of squared residuals:


$$\underset{{\hat{\beta }}_{0},{\hat{\beta }}_{1},\ldots ,{\hat{\beta }}_{p}}{\min}\sum_{i=1}^{n}{\left(y_{i}-{\hat{y}}_{i}\right)}^{2},$$

where ${\hat{y}}_{i}$ is the fitted value for observation *i*. In matrix form, the estimator is given by:


$$\hat{\beta }={\left(X^{⊤}X\right)}^{-1}X^{⊤}y,$$

where **X** is the design matrix, **y** is the response vector, and $\hat{\beta }$ is the vector of estimated coefficients. The formulation and estimation are based on standard theory for linear models as described in Chambers (1992).

## 3 Results and discussion

### 3.1 Strain *in vitro* characterization

The selection of *Bacillus* strains from the Active Germplasm Bank of Multifunctional and Phytopathogenic Microorganisms at Embrapa Maize and Sorghum focused on laboratory tests to assess their ability to grow *in vitro* under high osmotic potential conditions (**Table 1**). This included evaluating colony growth in a medium with high sorbitol concentration and low water activity. Additionally, the selected strains were examined for mechanisms that may help mitigate plant drought stress, such as the ability to exude biofilm and exopolysaccharides. Some characteristics associated with growth promotion of roots and canopy, including the synthesis of indole-3-acetic acid (IAA) and siderophores, were also evaluated. Based on the results obtained from strains 5D5 (*B. velezensis*), 6E9 (*B. velezensis*), and 1A11 (*B. subtilis*), the next step was to evaluate the behavior of these three strains in a biological system involving both plant and soil in a controlled growth environment. For this purpose, soybean was selected due to its agronomic significance and because it is a crop with sensitivity to moderate and extreme variations in temperature and soil humidity—conditions that are commonly observed in many agricultural systems in Brazil.

**Table 1:** Plant-growing promotion attributes of three selected *Bacillus* strains.

Strain	Species identification	Sorbitol growing		Aw	EPS	BIO	SID	IAA (*µ*g mL^−1^)
		520 g L^−1^	780 g L^−1^					
1A11	*B. subtilis*	+	–	0.897	+	–	+	14.19^b^
5D5	*B. velezensis*	+	+	0.807	+	+	+	29.12^a^
6E9	*B. velezensis*	+	+	0.807	+	+	+	16.42^b^

+indicates positive activity; – indicates absence of activity.

Aw, water activity; EPS, Exopolysaccharide; BIO, Biofilm; SID (carboxylate), Siderophore; IAA, Indole-3 acetic acid.

Values are the mean of three replicates. Means followed by the same letter do not differ significantly at the 5% level by the Scott-Knott test.

The identification of microorganisms that can thrive in low humidity and simultaneously produce biologically active substances holds significant promise for various applications, including enhancing plant tolerance in marginal environments. The bacterial strains were initially isolated from arid soils and the strains capable of growing in culture medium containing sorbitol at concentrations of 520 g L^−1^ and/or 780 g L^−1^ proceeded to the next stages of selection and evaluation. Further, a series of screenings was carried out to identify multifunctional mechanisms for plant growth-promoting (PGP) related to drought stress tolerance, particularly those related to exopolysaccharide (EPS) and biolfim production and capacity of growing in a low water activity. Water activity (aw) refers to the amount of water available for biological processes, and a value of 0.807, where 5D5 and 6E9 strains could grow, indicates a reduced moisture availability in the soil. For example, most microbes cease cell division below 0.9, and few prokaryotic strains have been shown to continue cell division at aw smaller than 0.755 (Amina and Lotfi, 2024). However, the strains 5D5, 1A11, and 6E9 screened in water-restricted culture media were efficient in producing EPS *in vitro*.

EPS are high-molecular-weight carbohydrates attached to the external surface of bacteria, related to the formation of biofilms and the attachment of bacterial cells to surfaces, including plant roots and soil particles. Since EPS are hydrated compounds, including 97% water in a polymeric matrix, they can enhance growth and ensure the survival of plants under drought stress (Vu et al., 2009; Nocker et al., 2012). In addition, they can protect the plant against desiccation due to the formation of hydrophilic biofilms on the root surface (Rossi et al., 2012). Although EPS is the main constituent of the biofilm, the 1A11 strain did not show biofilm production *in vitro*. Since the presence of EPS and biofilm are closely related, the difference between the number of bacteria producing these two compounds can be explained by the need for other substances to be present for biofilm formation, such as extracellular proteins or molecules related to hydrophobicity, which makes it more or less soluble (Vlamakis et al., 2013; Zhang et al., 2015). The production of EPS by bacteria is important because it increases soil permeability and aggregation, maintaining high water potential near the roots (Alami et al., 2000), which facilitates the diffusion of nutrients and the mass flow of soluble substances, increasing plant absorption and therefore, promoting the mitigation of water deficit. Inoculation experiments of maize seeds with EPS-producing bacterial strains, in combination with their respective purified EPS, resulted in increased soil moisture content, plant biomass, root and shoot length, and leaf area (Zhang et al., 2015). The microenvironments provided by EPS retain water and dehydrate more slowly than the surrounding environment, thus protecting the rhizosphere from desiccation.

The *Bacillus* isolates in this study also produced Indole-3 acetic acid (IAA), ranging from 14.19 to 29.12 µg mL^−1^. IAA is a member of the group of phytohormones normally considered the most important native auxins. It has been frequently reported as an important mechanism for plant growth promotion, improving root size and distribution, and ensuring an increase in nutrient uptake from the soil. The production of IAA by bacteria differs between species and can vary between strains, but it is also influenced by culture conditions, availability of vitamins, salts, oxygen, pH value, temperature, growth rate, and available nitrogen source (Duca et al., 2014), while the tryptophan, an amino acid used as an IAA precursor, can be provided by the plant roots.

### 3.2 Comparison of treatment means in the greenhouse environment

To assess the ability of *B. velezensis* strains 5D5 and 6E9, and *B. subtilis* strain 1A11 to enhance growth in soybean plants in comparison to a control inoculated only with *B. japonicum*, an experiment using pots were carried out in a greenhouse with controlled environmental conditions. The experiments consisted of dose-range of the three inoculants, evaluating the effects of 1, 2, 3, and 4 mL of inoculant per kg of seeds. The experiment was divided into two groups: one with adequate moisture for optimal plant development and the other subjected to water restriction, simulating a drought episode.

**Table 2** displays the response of soybean plants in terms of Shoot Fresh Weight (SFW), Shoot Dry Weight (SDW), Root Dry Weight (RDW), Number of Nodules per Root (NNR), and Mass of Functional Nodules per Root (MFNR) under irrigated and non-irrigated conditions. In both conditions, it was observed that the average treatment effects for certain inoculant doses were higher than the average of the control groups (T13 and T26 for irrigated and non-irrigated, respectively).

**Table 2:** Mean values of shoot and root biomass, number of nodules, and mass of functional nodules under irrigated and non-irrigated conditions.

Treatment	Condition	Strain	Dose (mL·kg^−1^seed)	SFW	SDW	RDW	NNR	MFNR
T1	Irrigated	1A11	1	54.89b	10.69b	2.03b	53b	0.99b
T2	Irrigated	1A11	2	55.86b	11.60b	2.51b	79a	1.89a
T3	Irrigated	1A11	3	52.14b	9.97b	2.33b	68a	1.83a
T4	Irrigated	1A11	4	72.07a	15.59a	3.93a	67a	0.94b
T5	Irrigated	6E9	1	58.86a	11.64b	2.80a	57b	1.42a
T6	Irrigated	6E9	2	67.98a	13.71a	2.85a	73a	1.50a
T7	Irrigated	6E9	3	63.89a	12.92a	2.81a	77a	1.63a
T8	Irrigated	6E9	4	63.04a	13.00a	2.95a	86a	1.75a
T9	Irrigated	5D5	1	65.48a	13.56a	3.11a	53b	1.40a
T10	Irrigated	5D5	2	62.99a	12.93a	2.74a	46b	1.00b
T11	Irrigated	5D5	3	52.43b	9.82b	2.64a	58b	0.88b
T12	Irrigated	5D5	4	44.30c	9.01b	2.27b	80a	1.28a
T13	Irrigated	Control	–	56.21b	11.71b	2.53b	81a	1.37a
T14	Non-irrigated	1A11	1	31.95c	7.44c	1.61a	48a	0.31b
T15	Non-irrigated	1A11	2	30.77c	7.13c	1.88a	20b	0.25b
T16	Non-irrigated	1A11	3	29.09c	6.86c	1.59a	37a	0.27b
T17	Non-irrigated	1A11	4	38.03a	8.71a	1.75a	24b	0.39a
T18	Non-irrigated	6E9	1	35.10b	7.86a	1.94a	37a	0.65a
T19	Non-irrigated	6E9	2	28.33c	6.59c	1.64a	25b	0.23b
T20	Non-irrigated	6E9	3	29.07c	6.98c	1.52a	21b	0.19b
T21	Non-irrigated	6E9	4	37.66a	8.85a	2.07a	35a	0.56a
T22	Non-irrigated	5D5	1	36.42a	7.90a	1.88a	48a	0.55a
T23	Non-irrigated	5D5	2	33.73b	7.85a	1.73a	45a	0.42a
T24	Non-irrigated	5D5	3	33.85b	8.12a	1.95a	41a	0.26b
T25	Non-irrigated	5D5	4	33.71b	8.20a	1.91a	34a	0.28b
T26	Non-irrigated	Control	–	27.38c	6.48c	1.77a	40a	0.27b

SFW, Shoot fresh weight; SDW, Shoot dry weight; RDW, Root dry weight; NNR, Number of nodules per root; MFNR, Mass of functional nodules per root. Different letters in the same column indicate statistically significant differences according to the Scott-Knott test at (p ≤ 0.05).

Under irrigated conditions (T1 to T13), treatment T4 (1A11, 4 mL·kg^−1^) exhibited the highest mean shoot fresh weight (SFW), not differing significantly from treatments T5 (6E9, 1 mL·kg^−1^), T6 (6E9, 2 mL·kg^−1^), T7 (6E9, 3 mL·kg^−1^), T8 (6E9, 4 mL·kg^−1^), T9 (5D5, 1 mL·kg^−1^), and T10 (5D5, 2 mL·kg^−1^). All these treatments outperformed the non-inoculated control (T13). A similar pattern was observed for shoot dry weight (SDW) and root dry weight (RDW), where the same treatments, except T5 for SDW, showed significantly higher values compared to the control. In contrast, no significant improvements were observed in the number of nodules (NNR) and the mass of functional nodules (MFNR) in inoculated treatments compared to the control (T13).

Under non-irrigated conditions (T14 to T26), for the variable shoot fresh weight (SFW), the treatments T17 (1A11, 4 mL·kg^−1^), T21 (6E9, 4 mL·kg^−1^), and T22 (5D5, 1 mL·kg^−1^) exhibited the highest shoot fresh weight (SFW) means, significantly surpassing the non-inoculated control (T26). Similarly, treatments T18 (6E9, 1 mL·kg^−1^), T23 (5D5, 2 mL·kg^−1^), T24 (5D5, 3 mL·kg^−1^), and T25 (5D5, 4 mL·kg^−1^) showed intermediate performance, but still significantly higher than the control. For shoot dry weight (SDW), treatments T17 (1A11, 4 mL·kg^−1^), T18 (6E9, 1 mL·kg^−1^), T21 (6E9, 4 mL·kg^−1^), and all doses of 5D5 (T22 through T25) recorded superior means compared to the control. In contrast, no significant differences were observed for root dry weight (RDW), suggesting that root development was uniformly constrained under water-limited conditions. Regarding the number of nodules (NNR), none of the treatments outperformed the control. However, for the mass of functional nodules (MFNR), treatment T18 had the highest mean, statistically comparable to T17, T22, and T23, all of which were significantly superior to the control.

The results revealed a strain- and dose-dependent effect of inoculation on plant growth and nodulation parameters, with notable distinctions between irrigated and non-irrigated conditions. Under irrigation, strains 1A11 and 6E9 significantly enhanced shoot fresh weight (SFW) and the mass of functional nodules per root (MFNR), particularly at moderate to high doses. Strain 1A11 reached the highest SFW (72.07 g) at 4 mL·kg^−1^, while 6E9 showed consistent performance across doses. In contrast, strain 5D5 exhibited reduced performance at higher doses, suggesting a possible inhibitory effect, which may be attributed to microbial competition or metabolic burden on the host plant (Etesami, 2025). It is also important to highlight that each microbial strain possesses distinct mechanisms for promoting drought tolerance, such as the production of phytohormones, enzymes, and biofilm formation. Additionally, the applied dose influences the number of viable cells and spores available to colonize the seed, thereby affecting both the survival and colonization efficiency of the inoculant. Notably, very high or very low inoculum doses may not necessarily benefit the plant, as the concentration of bioactive compounds, such as phytohormones, may become either excessive or insufficient, potentially leading to suboptimal plant responses (Etesami, 2025).

Under non-irrigated conditions, although the overall performance of inoculated plants was reduced compared to irrigated treatments, certain inoculant-strain combinations still demonstrated potential to confer drought resilience. For instance, 6E9 at 1 mL·kg^−1^ improved SFW (35.10 g) and MFNR (0.65 g) relative to the non-inoculated control (27.38 g and 0.27 g, respectively), and 5D5 at the same dose yielded the highest SFW (36.42 g) under drought. These results suggest that low-dose inoculation with well-adapted strains can mitigate drought stress effects, likely through improved symbiotic efficiency and modulation of root architecture, as previously documented in studies on plant-microbe interactions under water-limited conditions (Timmusk et al., 2014; Ngumbi and Kloepper, 2016; Vurukonda et al., 2016). In contrast, 1A11 enhanced SFW (38.03 g) and SDW (8.71 g), relative to the non-inoculated control under drought condition (27.38 g and 6.48 g, respectively).

Among the evaluated variables, SFW, SDW, and MFNR were the most positively responsive to inoculation, highlighting their utility as indicators of inoculant performance under contrasting moisture regimes. These findings reinforce the importance of selecting compatible strain-dose combinations and support the strategic use of plant growth-promoting bacteria to enhance legume productivity in both irrigated and drought-prone environments.

### 3.3 The impact of field weather on soybean yield

Abrupt and extreme climate variations have become one of the main challenges for agricultural practices, particularly in Brazil, a tropical country with a strong agricultural tradition. Regular planting and cultivation seasons are changing due to shifts in rainfall patterns, as well as variations in average maximum and minimum temperatures (Dos Santos et al., 2023). These changes increase the level of risk and make agricultural activities more unpredictable.

To mitigate these effects and identify their occurrence, farmers are adopting technological alternatives as part of their strategy to reduce losses and ensure economic returns. In this context, microbiological inoculants that improve plant resilience during mild to moderate water deficits—such as the bacterial strains evaluated in this study—are fundamental for maintaining crop grain yield. Some products are already available on the market, including those aimed at increasing phosphorus solubilization (de Oliveira-Paiva et al., 2024) and microbial-based solutions to reduce the effects of drought (Kavamura et al., 2013).

Additionally, incorporating technologies like remote sensing with time-series satellite or drone images enables producers to track in advance the environmental impacts on crop development. This allows them to take proactive palliative or corrective measures.

**Figure 1:**
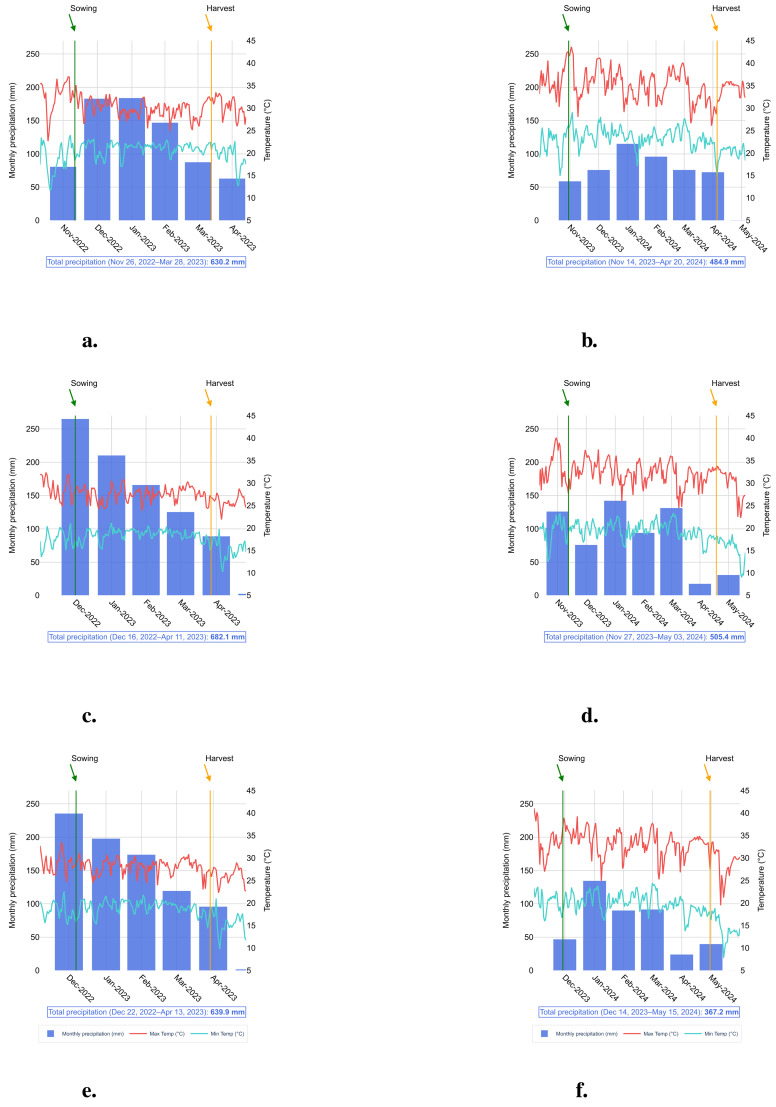
Monthly accumulated precipitation (mm), maximum temperature (°C), and minimum temperature (°C) during the experiment period. Locations, seasons and precipitation: **(a)** Birigui, season 2022/2023 and total accumulated precipitation of 630.2 mm, **(b)** Birigui, season 2023/2024 and total accumulated precipitation of 484.9 mm, **(c)** Itapira, season 2022/2023 and total accumulated precipitation of 682.1 mm, **(d)** Itapira, season 2023/2024 and total accumulated precipitation of 505.4 mm, **(e)** Piracicaba, season 2022/2023 and total accumulated precipitation of 639.9 mm, and **(f)** Piracicaba, season 2023/2024 and total accumulated precipitation of 367.2 mm.

During the field-trials with soybeans, significant variations were observed in rainfall patterns and temperature peaks (**Figure 1**). In the 2022–2023 season, rainfall was more regularly distributed and of a greater volume compared to the 2023–2024 season. The maximum temperatures recorded during 2022–2023 generally stayed below 35°C, whereas during 2023–2024, temperatures frequently exceeded 40°C, negatively affecting the development of soybean crops.

**Figure 2:**
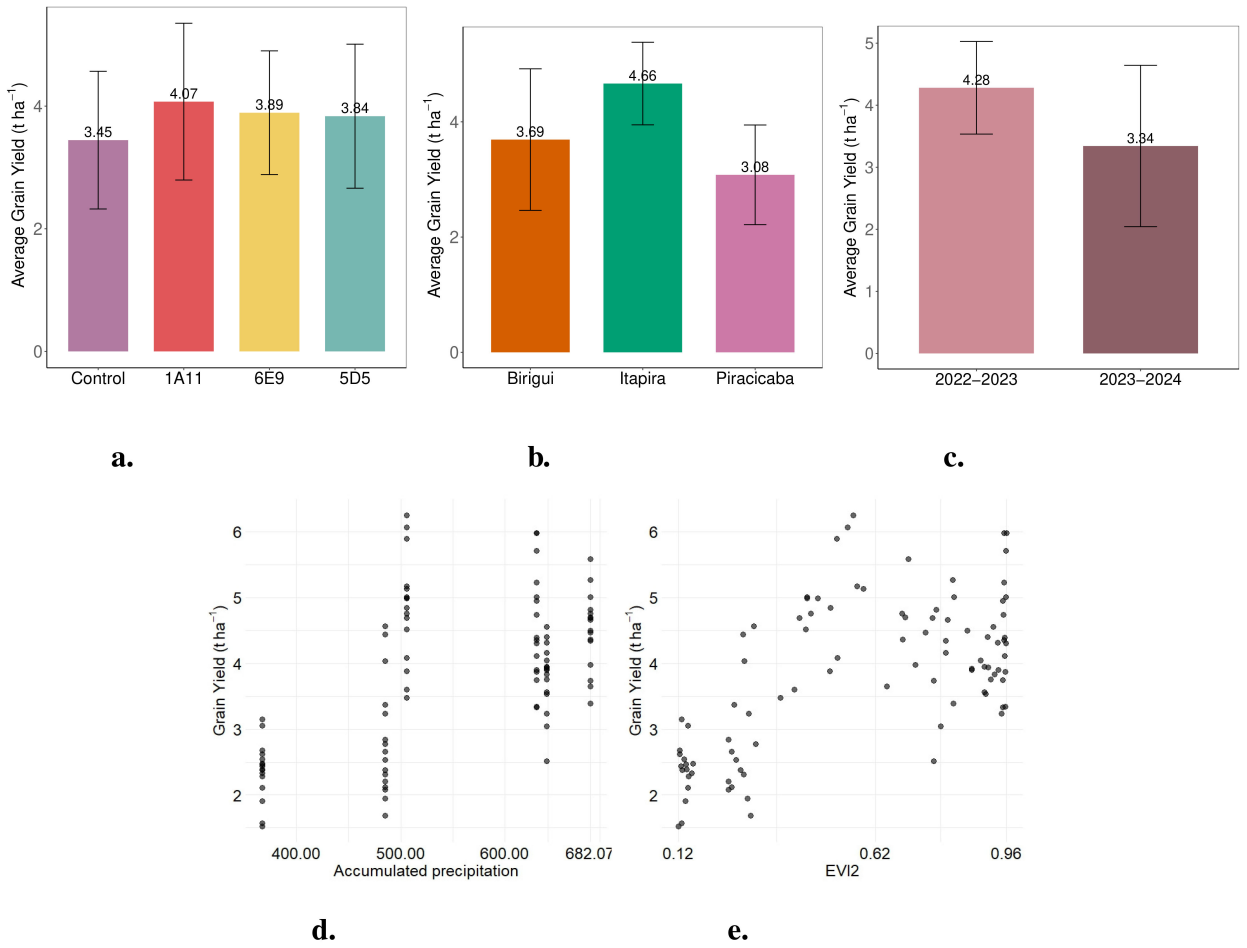
Bar plots of average grain yield (t ha^−1^) from the field trials: **(a)** by treatment, which received a dose of 3 mL·kg^−1^ of seeds, **(b)** by location, and **(c)** by crop year (2022–2023 and 2023–2024). Scatter plots from the field experiment: **(d)** cumulative precipitation and **(e)** mean EVI2 during the crop growth period versus grain yield (t ha^−1^).

As a result, the 2022–2023 harvest yielded an average of nearly one ton more than the 2023–2024 harvest average across the three locations involved in the study (**Figure 2c**). This clearly indicates the detrimental effects of climate extremes on soybean production. The analysis also revealed that Itapira had slightly higher average grain yield than Birigui and Piracicaba, likely due to better rainfall averages, especially durin7g the 2023–2024 season (**Figure 2b**). Beyond climate variations across locations (**Figure 1**), the differences in environmental conditions—particularly soil physicochemical properties—and the distinct genetic backgrounds of the three soybean varieties may also influence the results. This is worth mentioning that the three soybean varieties utilized in this study are categorized into maturity groups ranging from 6.1 (‘NS 5933 iPro’) and 6.4 (‘BMX Nexus i2X’) to 6.8 (‘Monsoy 6601 i2X’). Therefore, these varieties are suitable for the southeastern region of Brazil, where the field trials were carried out, and it also provided a wider planting window for the locations. This flexibility allowed to plant later in the season, exposing the crops to a period of lower rainfall and reduced soil humidity, and thus evaluate the effect of water shortage on the *Bacillus*/soybean crop association under conditions that are appropriate to water deficit.

### 3.4 Descriptive analysis of field experiments

**Figure 2a** shows that microbial strain 1A11 achieved the highest soybean yield across the two growing seasons and three tested locations, producing an average of 620 kg ha^−1^ more than the control (an 18% increase). Strains 5D5 and 6E9 also led to yield improvements over the control, with 5D5 yielding 390 kg ha^−1^ (11.3% increase) and 6E9 yielding 440 kg ha^−1^ (12.8% increase). These results suggest that the application of these treatments resulted in yield gains relative to the control.

Among the locations, Itapira achieved the highest average yield (4.66 t ha^−1^), followed by Birigui (3.69 t ha^−1^) and Piracicaba (3.08 t ha^−1^), as shown in **Figure 2b**. Furthermore, significant differences in yield were observed between the two growing seasons, with 2022–2023 (4.28 t ha^−1^) outperforming 2023–2024 (3.34 t ha^−1^), as shown in **Figure 2c**. According to **Figure 2**, increased cumulative precipitation (**Figure 2d**) and higher EVI2 (**Figure 2e**) values are associated with indications of greater soybean grain yield, suggesting that these variables may have a positive influence on crop yield.

**Table 3** summarizes the mean and standard deviation of grain yield (t ha^−1^) and the EVI2 index for different locations, treatments, and years. The data cover two seasons (2022–2023 and 2023–2024) and three locations: Birigui, Itapira, and Piracicaba. For each location and year, values for four treatments—1A11, 5D5, 6E9, and control—are provided. In 2022–2023 harvest, grain yield means ranged from 3.58 t ha^−1^ in the control treatment at Birigui to 5.14 t ha^−1^ in treatment 1A11 at the same location. EVI2 values were relatively stable across all treatments and locations in 2022–2023 season, with mean values close to 0.95. In 2023–2024 harvest, a noticeable decrease in grain yield was observed at most locations and treatments, particularly at Piracicaba, where the control treatment showed the lowest grain yield (1.78 t ha^−1^). EVI2 values remained consistent across treatments, with some variations, especially in Itapira and Birigui. These results reflect both grain yield levels and vegetation dynamics (as indicated by EVI2) across the two seasons.

**Table 3:** Mean and standard deviation of soybean grain yield (t ha^−1^) and Enhanced Vegetation Index 2 (EVI2) by location, treatment, and year under field conditions.

Location	Treatment	2022–2023				2023–2024			
		^1^Soybean grain yield Mean	^2^± SD	EVI2 Mean	^2^± SD	^1^Soybean grain yield Mean	^2^± SD	EVI2 Mean	^2^± SD
Birigui	1A11 5D5 6E9 Control	5.14 5.00 4.51 3.58	0.94 0.70 0.50 0.29	0.95 0.95 0.95 0.95	0.00 0.00 0.00 0.01	3.10 2.41 3.27 2.52	1.20 0.58 1.14 0.28	0.30 0.28 0.27 0.25	0.03 0.02 0.02 0.01
Itapira	1A11 5D5 6E9 Control	4.62 4.67 4.56 4.13	0.84 0.69 0.17 0.51	0.81 0.70 0.78 0.74	0.02 0.02 0.07 0.07	5.20 4.72 4.58 4.84	0.94 0.75 1.22 0.23	0.51 0.51 0.47 0.47	0.04 0.09 0.08 0.03
Piracicaba	1A11 5D5 6E9 Control	3.89 3.57 3.88 3.82	0.59 0.29 0.12 0.91	0.85 0.91 0.90 0.87	0.06 0.02 0.03 0.07	2.50 2.64 2.56 1.78	0.13 0.36 0.34 0.28	0.13 0.14 0.14 0.13	0.01 0.02 0.01 0.01

^1^Soybean grain yield Mean: Average yield of soybean grains in t ha^−1^; ^2^SD, Standard deviation.

Additionally, **Table 3** highlights that treatments with strains 1A11, 5D5, and 6E9 performed better in terms of EVI2 compared to the control. However, in Birigui during the 2022–2023 season, all treatments showed the same average EVI2 values, which were close to the maximum of the EVI2 scale (0.95), suggesting potential saturation under these specific conditions.

The high EVI2 values in Birigui during 2022–2023 may be attributed to the greater canopy development of soybean plants in all treatment groups, compared to other locations or years. Environmental variations in open field conditions likely contributed to these discrepancies.

### 3.5 Statistical modeling of field experiments

Based on the multicollinearity analysis, the explanatory variable *Year* (*x_i_*_4_) was excluded due to its high VIF in relation to the other covariates. The final model, without this variable, was fitted to represent the relationship between the dependent variable *y_i_* and the selected explanatory variables. The model equation is given by:


$$y_{i}=\beta_{0}+\beta_{1}b_{i1}+\beta_{2}b_{i2}+\beta_{3}b_{i3}+\beta_{4}t_{i1}+\beta_{5}t_{i2}+\beta_{6}t_{i3}+\beta_{7}l_{i1}+\beta_{8}l_{i2}+\beta_{9}x_{i5}+\beta_{10}x_{i6}+\epsilon_{i}.$$

This revised model, excluding the variable with high multicollinearity, results in a more robust and precise selection of explanatory variables.

The results of the statistical model (**Table 4**) indicated that treatments 1A11, 5D5, and 6E9 showed significant differences compared to the control (represented by the intercept), with statistical significance determined by *p*-values less than 0.10, however, these treatments did not differ from each other (**Table 5**). These findings highlight the efficacy of strains 1A11, 5D5, and 6E9 in enhancing soybean development under non-irrigated, open-field conditions. The observed grain yield gains were statistically significant compared to the not inoculated control, underscoring the potential of microbial inoculants to improve crop performance even in challenging agricultural environments.

**Table 4:** Estimated coefficients for the fitted model using field experiment data.

Effect	Parameter	Estimate	^1^SE	*p*-value
Intercept	*β*_0_	4.877	0.721	*<* 0.001
Block 2	*β*_1_	0.024	0.190	0.901
Block 3	*β*_2_	0.111	0.190	0.561
Block 4	*β*_3_	0.230	0.190	0.229
Treatment 1A11	*β*_4_	0.526	0.190	0.006
Treatment 5D5	*β*_5_	0.333	0.190	0.082
Treatment 6E9	*β*_6_	0.380	0.190	0.048
Location Itapira	*β*_7_	1.205	0.175	*<* 0.001
Location Piracicaba	*β*_8_	-0.564	0.170	0.001
Accumulated Precipitation	*β*_9_	-0.0077	0.0018	*<* 0.001
EVI2	*β*10	4.451	0.619	*<* 0.001

^1^SE, Standard Error.

2 SE: Standard Error.

**Table 5:** Results of multiple comparisons of treatment considering regression model.

Hypotheses *H*_0_	Estimate	^1^SE	*p*-value
1A11 - 5D5	-0.145	0.189	0.443
1A11 - 6E9	-0.192	0.189	0.312
5D5 - 6E9	0.046	0.189	0.805

^1^SE, Standard Error.

Regarding location, Itapira showed significantly higher average grain yield compared to the other sites, as illustrated in **Figure 2b**. The bar plot indicates that the mean yield in Itapira was greater than in other locations, particularly when compared to Birigui. This difference may reflect environmental and management variability between locations and the availability of rainwater throughout the crop cycle, with the rainfall regime being more favorable in some of the locations where the tests were carried out than in others (**Figure 1**).

As for cumulative precipitation, the model results indicate that this variable was statistically significant. However, excessive precipitation appears to be associated with a slight reduction in soybean grain yield. This trend is evident in **Figure 2d**, which shows a small decrease in grain yield at the highest levels of accumulated rainfall during the early stages of the experiment.

Finally, the EVI2 index showed a significant positive effect on grain yield: according to the fitted model, for each one-unit increase in EVI2, soybean yield increased by an average of 4.45 t/ha. It is worth noting that, as the crop approaches its final growth stages, a natural decline in vegetation indices is expected due to plant maturation and senescence.

**Figure 3:**
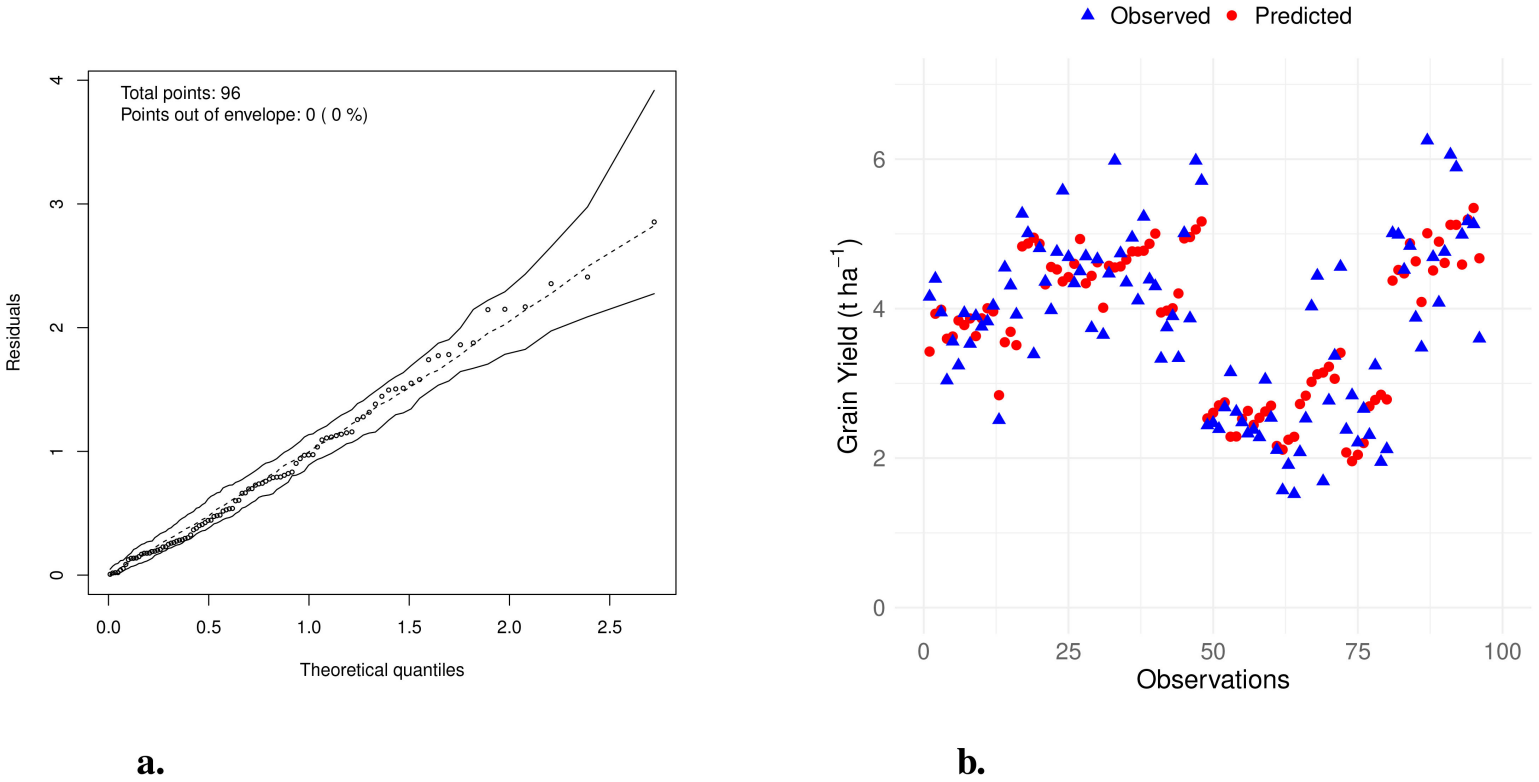
**(a)** Half-normal plot with simulated envelope for the regression model. **(b)** Observed vs. predicted values from the multiple linear regression model for soybean grain yield.

The half-normal plot (Moral et al., 2017) with simulated envelope (**Figure 3a**) showed that all standardized residuals fell within the envelope. This suggests that the linear model provides an adequate fit to the data, with residuals behaving as expected under the assumptions of normality and homoscedasticity.

The Shapiro-Wilk test yielded *W* = 0.9898 with a *p*-value of 0.6756, and the Breusch-Pagan test produced *BP* = 12.508 with *df* = 10 and a *p*-value of 0.2525. Both tests were non-significant, indicating that the residuals are normally distributed and exhibit constant variance, thus supporting the adequacy of the linear model.

Additionally, the model achieved a coefficient of determination (*R* ^2^) of 0.71, indicating that approximately 71% of the variability in soybean grain yield was explained by the included explanatory variables.

**Figure 3b** illustrates the comparison between observed and predicted grain yield values obtained from the multiple linear regression model. The close alignment between observed and predicted points demonstrates that the model effectively captures the variability in soybean grain yield. In the figure, blue triangles represent the observed values, while red circles indicate the predicted values. The overlapping of these points reflects the natural variability in the data and supports the model’s accuracy in predicting grain yield.

Research utilizing remote sensing tools to study microbiological inoculants is surprisingly limited in the literature, revealing a significant gap but also presenting opportunities to improve the assessment of these inoculants’ effectiveness on agronomic crop performance. For example, de Souza et al. (2022) conducted a study on common beans, using UAVs to evaluate and monitor the biofertilization process. They applied VIs to map spectral changes in common beans after the application of phosphate-solubilizing bacteria in a remote and non-destructive manner. Similarly, Santos et al. (2024) investigated phosphate-solubilizing bacterial inoculants in sugarcane crops developed a predictive statistical model that accurately forecasted sugarcane grain yield in fields treated with these microbiological inoculants, producing predictions months ahead of harvest that closely aligned with actual field data.

## 4 Conclusion

This study demonstrates that *B. velezensis* (5D5, 6E9) and *B. subtilis* (1A11) microbiological inoculants significantly enhance soybean resilience and grain yield under drought stress, validating their role as scalable, sustainable tools for climate-smart agriculture. The *Bacillus* strains 1A11, 5D5 and 6E9, isolated from arid soils, showed multifunctional mechanisms for plant growth-promoting (PGP) related to drought stress tolerance, particularly those related to exopolysaccharide (EPS) and biolfim production and capacity of growing in a low water activity. Greenhouse trials revealed that doses of microbial biostimulants above 2 mL Kg^−1^ of seed application were able to increase biomass accumulation, with inoculated plants under drought stress surpassing non-inoculated controls. Field-trials across three Brazilian agroclimatic zones under non-irrigated conditions further confirmed the grain yield superiority of *Bacillus* of 1A11, 5D5 and 6E9 compared to the control treatment without seed inoculation. These results position microbial biostimulants as critical alternatives to synthetic inputs, aligning with Brazil’s strategic priorities to reduce agrochemical dependency while safeguarding food security. The integration of satellite and UAV-derived EVI2 allowed for precise, non-destructive monitoring of treatment effectiveness, ensuring a consistent overlap between field data and model predictions. This dual assessment framework—combining agronomic metrics with remote sensing—provides a novel, cost-effective strategy for real-time crop performance evaluation.

## Data Availability

The raw data supporting the conclusions of this article will be made available by the authors, without undue reservation.
